# Effects of ischemic pre-conditioning on electrically stimulated contractions

**DOI:** 10.1007/s00421-024-05577-1

**Published:** 2024-08-21

**Authors:** Ruben Allois, Pasquale Pagliaro, Ermini Leonardo, Silvestro Roatta

**Affiliations:** 1 Laboratory of Integrative Physiology, Department of Neuroscience, University of Torino, c.so Raffaello 30, 10125 Turin, Italy; 2 Department of Clinical and Biological Sciences, University of Torino, Turin, Italy

**Keywords:** Ischemic pre-conditioning, Electromyographic, Force, Hemodynamic variables, Electrically stimulated contraction

## Abstract

**Purpose:**

Ischemic pre-conditioning (IPC) offers protection against future ischemic events and may improve sports performance due to several mechanisms at local and systemic levels. This study investigates the local effects on muscle contractility in electrically induced muscle contractions, thus effectively excluding any uncontrolled change in the motor drive.

**Methods:**

Twenty-one subjects were divided into two groups: 12 subjects in the IPC group (3 × 5/5 min right arm ischemia/reperfusion; cuff pressure 250 mmHg) and 9 subjects in the SHAM group (same treatment at 20 mmHg). The adductor pollicis was contracted by supramaximal stimulation of the ulnar nerve with single pulses, trains of stimuli (5, 8, 10 and 12 Hz, 1-s duration) and bursts (4 pulses, 25 Hz), all separated by 5-s intervals. The stimulation sequence was delivered before and 15 and 30 min after IPC/SHAM treatment. The isometric contraction force, the superficial electromyographic signal, and tissue oxygenation were continuously monitored.

**Results:**

A significant force decrease in time was observed at 8, 10 (*p* < 0.01) and 12 Hz (*p* < 0.05) along with a decrease in half-relaxation time in single twitches and bursts (*p* = 0.01), regardless of treatment. This general time-related weakening was more marked in IPC than SHAM at 5-Hz stimulation. No effects were observed on the magnitude of the superficial electromyographic signal.

**Conclusion:**

Data indicate that IPC does not increase muscle force during electrically stimulated contractions, supporting the idea that IPC’s ergogenic effects are not due to increased muscle contractility.

## Introduction

Ischemic pre-conditioning (IPC) is a phenomenon where brief bouts of ischemia–reperfusion confer protection against prolonged ischemia and reperfusion injury (Pagliaro et al. 2001). First investigated in the cardiac muscle (Fox et al. 2018), IPC has later been applied to protect several tissues and organs (Morris et al. 2015) and to mitigate impairment of skeletal muscles related to contraction-induced ischemia (Incognito et al. 2016). Different mechanisms have been suggested to underlie the beneficial effects, including increased vascular endothelial function and reduced blood lactate, muscle damage and fatigue (O’Brien and Jacobs 2022).

IPC has also been documented to enhance sports performance (Bailey et al. 2012; Kjeld et al. 2014; Patterson et al. 2015; Salvador et al. 2016). IPC protocols typically involve unilateral or bilateral ischemic periods lasting 3 × 5 or 4 × 5 min applied to the upper and lower limbs (Bailey et al. 2012; Kjeld et al. 2014; Patterson et al. 2015). Improvement rates ranging from 1 to 8% (Salvador et al. 2016) have been reported across different sports disciplines including running (Bailey et al. 2012), cycling (Patterson et al. 2015) and static/dynamic apnea (Kjeld et al. 2014). In particular, an ergogenic effect of IPC has frequently been suggested based on experimental findings demonstrating increased muscle force capacity or increased endurance, such as augmented maximum voluntary knee extension in male subjects (Paradis-Deschênes et al. 2017).

However, there are conflicting results across studies, with some reporting no discernible effect. Doubts have also arisen regarding the consistency and practical significance of the observed effects (Souza et al. 2021). Also, no univocal explanation exists about the possible underlying mechanisms, as IPC may produce effects also on untreated organs (remote IPC) and changes at the systemic level in addition to local effects on treated muscles (Hausenloy and Lim 2012). In fact, different mechanisms have been proposed that may have a role in increasing sports performance. At the peripheral level, in indirectly or remotely affected muscles, there is evidence of decreased oxygen consumption (Allois et al. 2023), improvements in phosphocreatine resynthesis (Andreas et al. 2011) and improved endothelium-dependent vasodilation in humans (Kimura et al. 2007). At the same time, centrally originating changes in the motor drive have been hypothesized, as produced by group III and IV afferents from IPC-exposed muscles (Crisafulli et al. 2011) by humoral factors reaching the central nervous system via the blood stream (Hausenloy and Yellon 2008), as well as the placebo effect, to the extent that performance enhancements have sometimes been observed in response to a SHAM treatment, e.g., in resistance exercise (Marocolo et al. 2016). While some of these mechanisms may manifest only during resistance or fatiguing contractions, ergogenic effects have also been described in resting or non-fatigued conditions (Rodrigues et al. 2023; Telles et al. 2022b), but the possible occurrence of specific effects at the peripheral level was not investigated.

A reliable method to discriminate between central and peripheral mechanisms is to bypass the central motor drive and its possible uncontrolled changes by investigating electrically stimulated (ES) rather than voluntary muscle contractions. The electrically stimulated contraction has some disadvantage related to the possible discomfort provided to the subject and the non-physiological modality of contraction, due to the synchronous rather than asynchronous recruitment of motor units. On the other hand, it is an ideal model for the investigation of local muscle contractility while excluding central effects, provided that reflex motoneuronal activation is avoided (Maffiuletti 2010). To our knowledge, in the very few studies adopting this experimental model, the investigations were limited to single muscle twitches and failed to evidence significant IPC-induced changes (Halley et al. 2018, 2019a). However, small effects may be difficult to detect in single twitches, in terms of changes in peak force duration, while they may be best appreciated in sub-tetanic contractions or in response to short burst stimulation (Roatta and Farina 2011). However, electrical stimulation of a peripheral nerve also includes activation of afferent fibers from muscle spindles and other peripheral receptor, which may affect motoneuronal excitability and lead to the generation of additional EMG activity and the development of extra muscle force (Collins et al. 2002). This effect, which would produce undesired spurious changes in muscle force, can however be excluded by limiting the duration and the frequency of the stimulation (Maffiuletti 2010).

The present study aims to test the hypothesis that the ergogenic effect of IPC may result from a modulation of the contractile machinery. To this aim, we investigated the IPC effects on ES contractions of the adductor pollicis (AP), which included single pulses, short trains of stimuli at different frequencies and short bursts.

## Materials and methods

### Subjects and study design

Twenty-one subjects were enrolled: 12 subjects (age: 26 ± 9 years, weight: 71 ± 10 kg, height: 180 ± 1 cm; BMI: 22.6 ± 2.7 kg m^2^) in the IPC group, and 9 subjects (age: 25 ± 11 years, weight: 75 ± 9 kg, height: 181 ± 1 cm; BMI: 23.0 ± 2.8 kg m^2^) in the SHAM group (3 × 5/5 min pseudo ischemia/reperfusion; 20 mmHg). Two subjects took part to both groups. Participants were eligible for the study if they met the following criteria: healthy individuals with no prior history of medical conditions, non-smokers and those free from any medications that might affect the outcome measures. Participants were excluded if they had any neuromuscular impairments that would prevent them from completing the study procedures. The study was conducted in agreement with the principles of the Declaration of Helsinki (2000) and under the approval of the Ethics Committee of the University of Torino. The subjects gave written informed consent.

### Experimental setup

**Fig. 1:**
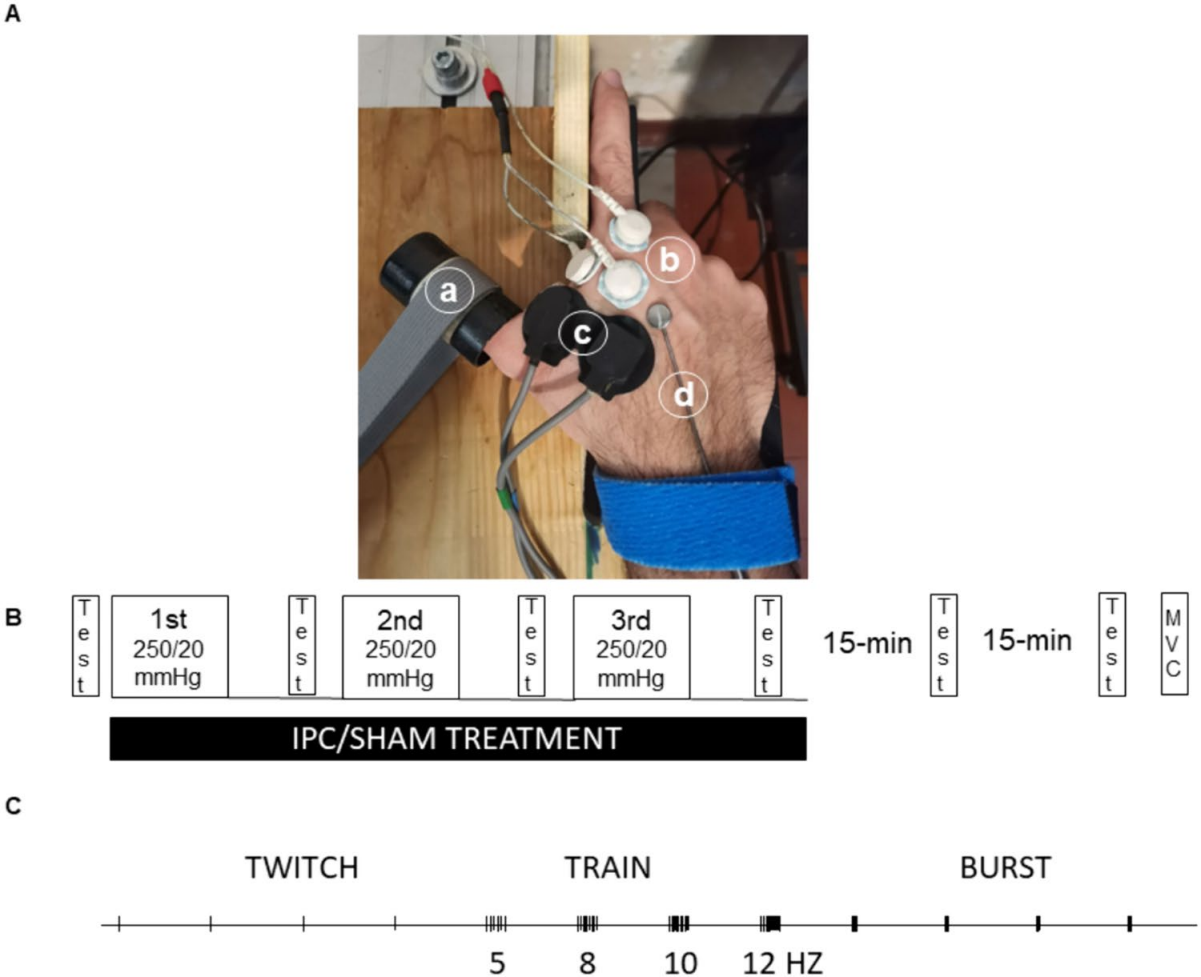
**A** Experimental setup. The right hand rests over a horizontal surface, blocked by a wrist band. A rigid band (a) connects the thumb to a load cell (not visible); one of the sEMG electrodes (b) is located on the medial end of the abductor pollicis, and a near-infrared spectroscopy probe (c) over its lateral end; a temperature probe (d) is also located on the hand dorsum. **B** Schematic representation of the experimental protocol. A sequence of electrical stimulations of the ulnar nerve (test) is delivered before, during and after the ischemic pre-conditioning (IPC) or placebo (SHAM) treatment. **C** Electrical stimulation sequence. The stimulation includes four single pulses (twitches), 1 s lasting trains of stimuli at 5, 8, 10 and 12 Hz, and, four bursts consisting of four pulses at 25 Hz, all separated by 5-s intervals

As can be observed in Fig. 1A, an arm rest was designed for implementing isometric stimulated contractions of the thumb adductor of the right hand. Note that the wrist is blocked by a belt, the leftward movement of the index finger is impeded by a wooden bar, the thumb is rigidly and perpendicularly connected to a load cell and the third, fourth and fifth fingers may freely move downward, so that their flexion, elicited by electrical stimulation of the ulnar nerve, does not displace other parts of the hand and interfere with the thumb adduction. The subject sat, with back and arms rested, elbows flexed at about 120 degrees and the shoulder adducted and neutrally rotated.

### Electrical stimulation protocol

**Fig. 2:**
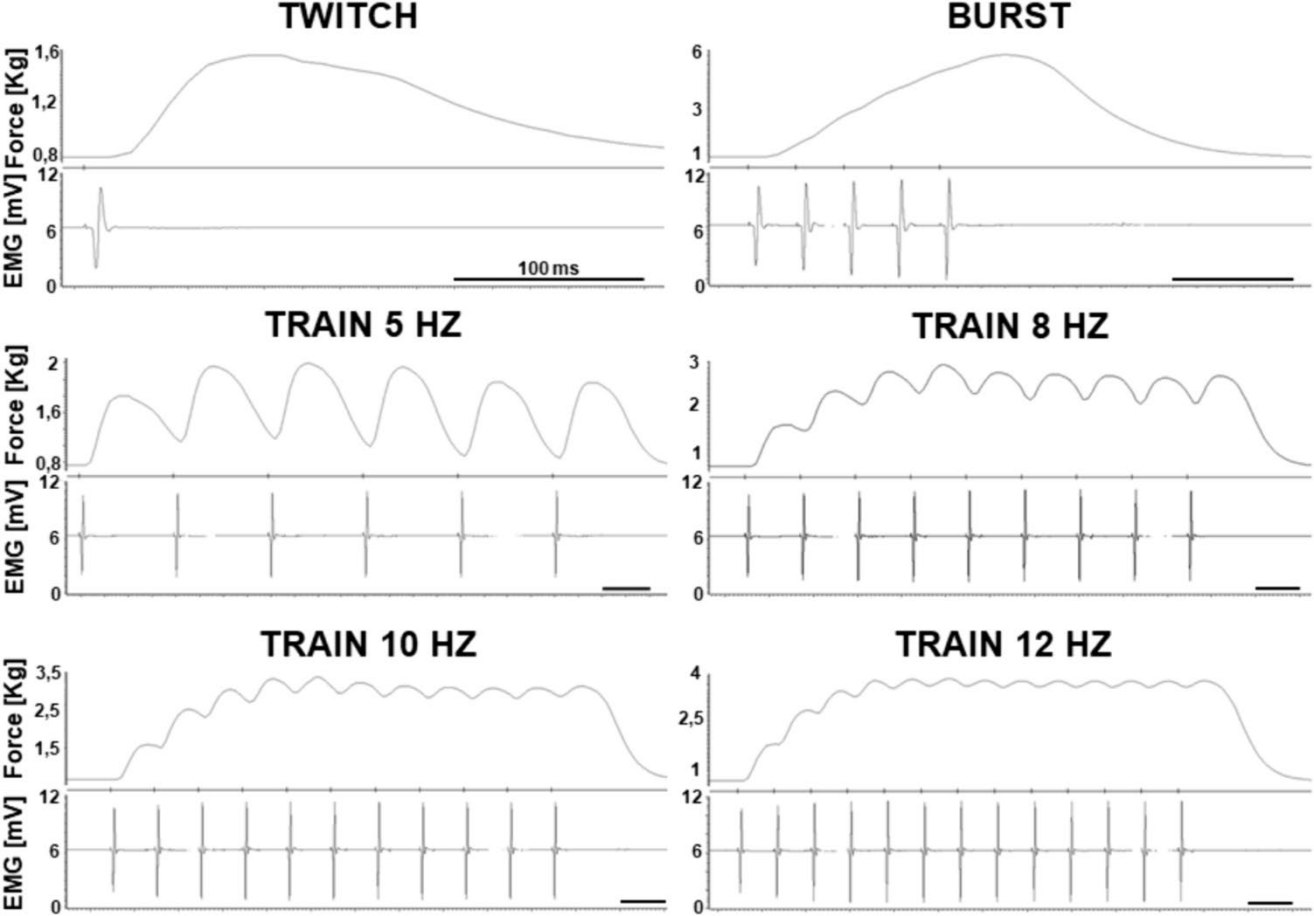
Force and sEMG signals. Force and electromyography (EMG) recordings from a representative subject of contractions evoked by the different patterns of stimulation: single twitch, burst and short train stimuli at different frequencies (5, 8, 10 and 12 Hz). The black horizontal bar in each plot represents a duration of 100 ms

A scheme of the experimental protocol is given in Fig. 1B. The subject was familiarized with the setup and invited to relax during the electrical stimulation protocol. To find the supramaximal intensity of stimulation, the subject received single pulses (1-ms duration) at increasing stimulation intensity, with steps of 5 mA, every 10 s, until both the force and the sEMG signal ceased to increase. That intensity level was further increased by 5 mA to set the supramaximal stimulation level (SSL), which was used for all subsequent stimulations. The stimulation sequence included: four single pulses (twitches), four 1-s lasting trains of stimuli at 5, 8, 10 and 12 Hz and four bursts, each consisting of five pulses at 25 Hz, all separated by 5-s intervals (Fig. 2). This stimulation sequence was delivered before, during the IPC/SHAM treatment (in-between pneumatic compressions, after 3 min of each deflation), and 15 min (POST-15) and 30 min (POST-30) after the treatment. The sequence and timing of stimuli were maintained absolutely identical and not randomized, so that any order effect should be similarly reproduced in all conditions and have no impact in the comparisons. Care was taken to maintain the arm at a constant temperature, with the help of an infrared lamp. After 2 min from the last electrical stimulation sequence, the maximum voluntary contraction (MVC) of the AP was measured as the maximum developed force (averaged over 0.5 s) in three attempts separated by 2-min rest intervals. The MVC measurement was performed at the end rather than at the beginning of the session to avoid the possible confounding effects of post-tetanic potentiation (Baudry and Duchateau 2007).

### IPC/SHAM treatment

The IPC treatment was delivered to the right arm using a pneumatic cuff (The Occlusion Cuff^®^ 8 × 75 cm, The Occlusion Cuff LTD, Somerset, UK). The brachial artery was occluded by rapidly inflating the cuff to 250 mmHg or to 20 mmHg according to the IPC or SHAM treatment for 5 min. This stimulus was repeated two other times, separated by 5-min rest intervals. Rapid cuff inflation and deflation was achieved by a custom system based on a PC-driven proportional valve (ITV1010, RTI Srl, Torino, Italy) (Ferraresi et al. 2019; Messere et al. 2018).

### Temperature, blood pressure and hemodynamic variables

During the protocol, the cutaneous temperature (TEMP) was continuously monitored from the back of the hand (TERZMI-I, Terzano & C Srl, Milano, Italy). Arterial blood pressure (ABP; diastolic ABPd and systolic ABPs) was measured before, 5 min and 30 min after the treatment through a digital sphygmomanometer (CNSystems, Medizintechnik GmbH, Graz, Austria). The non-invasive monitoring of muscle oxygenation was performed using near-infrared spectroscopy (NIRS) (NIRO-200X, Hamamatsu Photonics K.K, Shizuoka, Japan). Two NIRS probes, with an inter-optode distance of 3 cm, were positioned over the dorsal side of the AP muscle (see Fig. 1A) and over the anterior side of the forearm. The device measures the tissue oxygenation index (TOI), which represents the oxygen-saturated (hemoglobin + myoglobin) level in percentage, and the tissue hemoglobin index (THI), which represents the total (hemoglobin + myoglobin) concentration in arbitrary units. These parameters are based on the spatially resolved methodology, which focuses on the measurement in depth (muscle tissue) and, thus, it is little affected by hemodynamic changes occurring in the superficial cutaneous tissue layer (Messere and Roatta 2013, 2015).

### Electrical stimulation and EMG recording

Contraction of the AP was obtained by bipolar electrical stimulation of the ulnar nerve (DS7A, Digitimer Ltd in Welwyn Garden City, England) using two electrodes (Euro Ecg Eletrodes, Fiab Srl, Florence, Italy) located 5 and 7 cm proximal to the wrist. The other two electrodes were cut following the round shape (1 cm) and placed at a distance at 2 cm, located about 5 cm proximal to the wrist. Surface electromyographic activity of the AP was bipolarly recorded (QUATTRO, OT Bioelettronica Srl, Turin, Italy) with one electrode placed on the dorsal side of the AP, close to the NIRS probe and the other two placed over the second metacarpal bone. The skin was previously cleaned using an abrasive prepping gel.

### Force recording

The force developed by the electrically stimulated contractions of the AP was recorded by a load cell (DYLY-103-5kg, CaltSensor Ltd, Shanghai, China) and a dedicated amplifier (Forza-B, OT Bioelettronica Srl, Turin, Italy). The load cell was connected to the thumb by a rigid strap. The length of the strap was adjusted to develop a slight tension, of about 2 N, in the passive muscle.

All signals were digitally sampled (1401micro, CED, Cambridge, UK) at a sampling frequency of 1024 Hz, except for the surface electromyographic signal (sEMG), sampled at 2 kHz, and stored on PC for off-line analysis (Spike2, CED, Cambridge, UK). Data acquisition was continuously performed throughout the whole experimental session.

## Data analysis

For the analysis of the response to single and burst stimuli, the average response curve was first calculated from the second, third and fourth stimulation (the first stimulation, presenting high variability, was excluded from the analysis). From the average force–response curve, the following measurements were taken: the peak force (PF) was calculated as the difference between the peak and basal (pre-stimulation) level; the time to peak (PT) as the time elapsed from stimulation to achievement of peak force; the half-relaxation time (HRT) as the time required to reach 50% PF from the force peak.

The average curve (M-wave) was also calculated for the sEMG signal in response to single stimuli (width: 0.1 s; offset 0.03), and the peak-to-peak amplitude was extracted (AmpliPP).

As for the analysis of the 1-s train stimulations, from each contraction the following measurements were extracted from the last 0.5 s of stimulation: the mean force (MF), as the average force value reached with respect to baseline, and the magnitude of force pulsatility (PLS), as the average of the difference between maximum and minimum force, detected within single stimulation periods over the 0.5-s interval considered. The first 0.5 s, required to reach a steady force level, was excluded from the analysis.

The average values of ABPd, ABPs, TOI, THI and TEMP were measured over the 60 s preceding the stimulation before and 15 and 30 min after the IPC/SHAM treatment. All signal processing was performed with the acquisition and analysis software Spike2 and the measured values were collected in Excel sheets.

## Statistical analysis

Statistical analysis was performed using SPSS 27.0 (SPSS Inc., Chicago, Illinois, USA). The data sets were analyzed and the results are presented as mean ± standard deviation. *p* values less than 0.05 were considered significant. Normality of data was checked by Shapiro–Wilk’s test, while homoscedasticity was tested by Levene’s test. Subjects’ age, weight, height, BMI, MVC and SSL were statistically compared between groups (IPC vs. SHAM) using either Mann–Whitney *U* test or independent *t* test, depending on the results of normality and homoscedasticity tests. For each variable concerning the electrical stimulation response, i.e., ABPd, ABPs, TEMP, TOI and AmpliPP, a two-way mixed ANOVA was performed, to investigate the effect of both time (pre-, POST-15 and POST-30; within-subjects) and group (IPC and SHAM; between-subjects). THI, which is automatically normalized (set to 1) at the beginning of the recording, only the main effect of time was investigated. For each pattern of electrical stimulation and for each variable concerning the force response, i.e., PT, PF and HRT, a different two-way mixed ANOVA was performed, to investigate the effect of both time (pre-, POST-15 and POST-30) and group (IPC and SHAM). Then, if an interaction was present, a Tukey’s post hoc test was performed to analyze pairwise differences.

## Results

The IPC and SHAM groups did not differ in age, weight, height, BMI and muscle strength in terms of MVC (IPC: 9.6 ± 2.2 vs. SHAM: 10.5 ± 3.0 kg) and SSL (26.7 ± 4.4 vs. 28.3 ± 4.3 mA, n.s.). In addition, the two groups did not differ in terms of blood pressure (both ABPd and ABPs), and TOI (at AP), TEMP and AmpliPP were stable over time in both groups (Table 1). No evidence of extra-EMG activation, as could be elicited by high-frequency stimulation of afferent pathways (Collins et al. 2002), was detected in any of the recordings.

**Table 1:** Changes of different variables across time

Variable	Group	PRE	POST-15	POST 30	Main effects		
		Mean ± SD	Mean ± SD	Mean ± SD	Interaction	Time	Time*group
ABPd (mmHg)	IPC	75.9 ± 7.2	73.3 ± 8.3	73.1 ± 9.6	ns	ns	ns
	SHAM	76.8 ± 6.2	74.0 ± 7.3	75.9 ± 6.0	0.608	0.278	0.784
ABPs (mmHg)	IPC	120.3 ± 4.3	123.8 ± 11.4	120.8 ± 11.7	ns	ns	ns
	SHAM	121.8 ± 5.5	119.0 ± 6.2	121.0 ± 8.5	0.733	0.904	0.262
TOI (%)	IPC	65.13 ± 9.4	64.2 ± 9.4	64.8 ± 9.0	ns	ns	ns
	SHAM	66.1 ± 4.4	62.5 ± 5.78	63.1 ± 4.2	0.451	0.739	0.643
THI (%)	IPC	0.9 ± 0.1	0.8 ± 0.3	0.8 ± 0.3		ns	
	SHAM	1.1 ± 0.1	1.0 ± 0.4	0.9 ± 0.2		0.116	
TEMP (°C)	IPC	30.5 ± 2.6	31.1 ± 2.5	31.2 ± 2.5	ns	ns	ns
	SHAM	31.0 ± 2.1	31.1 ± 2.0	31.0 ± 1.9	0.919	0.241	0.348
AmpliPP (mv)	IPC	6.1 ± 2.8	5.9 ± 3.1	5.91 ± 3.0	ns	ns	ns
	SHAM	4.9 ± 3.1	5.0 ± 3.1	4.9 ± 3.2	0.136	0.180	0.613

Diastolic (ABPd) and systolic arterial blood pressure (ABPs), tissue oxygenation index (TOI), tissue hemoglobin index (THI), skin temperature (TEMP) and sEMG magnitude (AmpliPP) values collected before (PRE) and 15 min (POST-15) and 30 min (POST-30) after the IPC (ischemic pre-conditioning) or SHAM treatment. No significant differences between groups and between conditions and no significant interactions were detected in any of the variables

### Single twitches

**Fig. 3:**
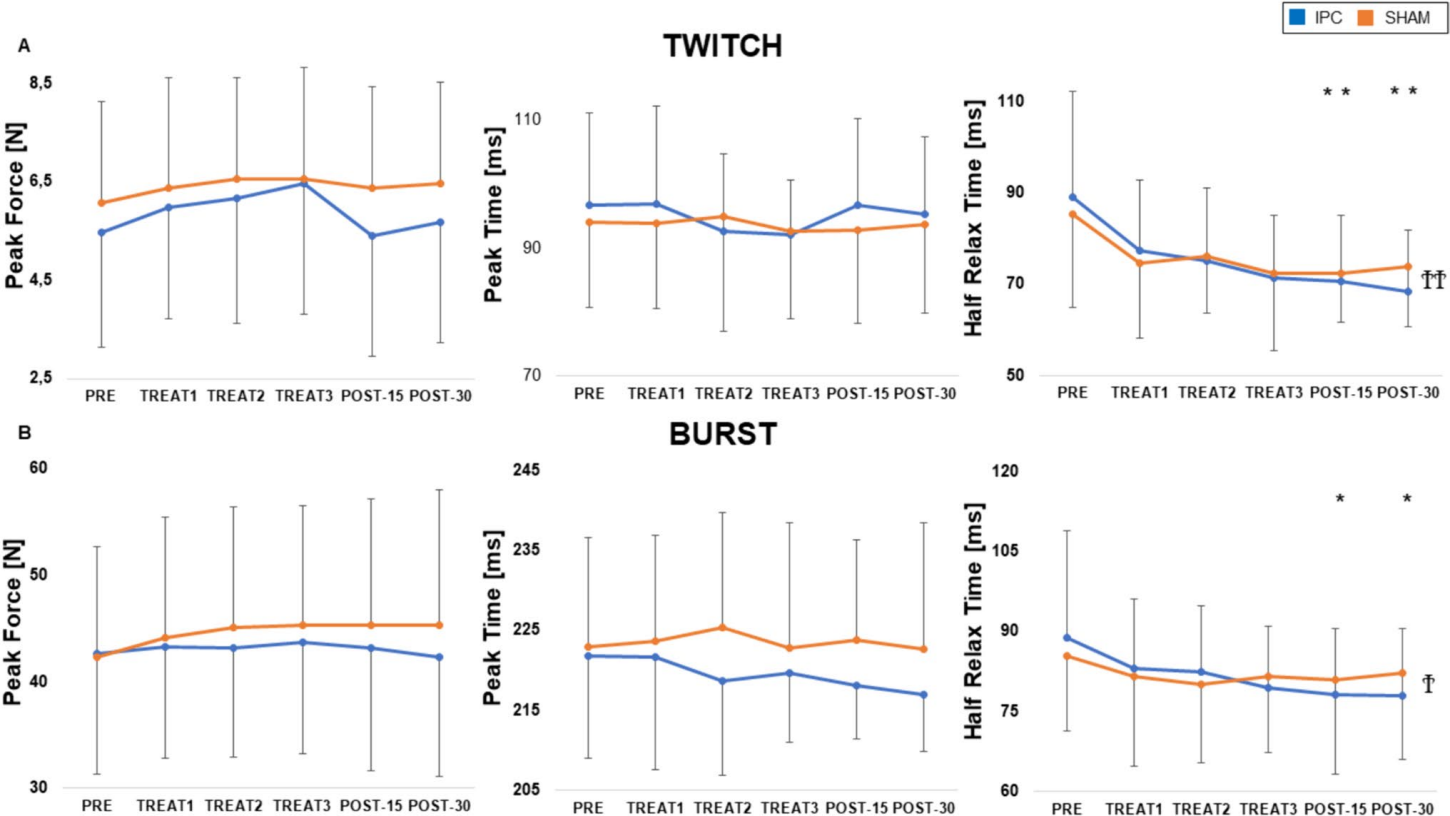
Twitch and burst. **A** Average effect of IPC (black) and SHAM (gray) treatments on the different parameters of the electrically stimulated single twitches (**A**) and bursts (**B**) at the different time points. ^Ϯ^ = main effect of time, *p* < 0.05; ^ϮϮ^ = main effect of time, *p* < 0.01; ^*^ = PRE vs. POST, *p* < 0.05; ^**^ = PRE vs. POST, *p* < 0.01

No main effect of treatment or time and no interaction between the two were observed on PT and PF. However, there was a significant effect of time (*p* < 0.01) on HRT, with no effect of treatment and no interaction, which was significantly decreased at POST-15 and POST-30. The results are graphically reported in Fig. 3A.

### Burst

The response to burst stimulation is depicted in Fig. 3B. No main effect of treatment or time and no interaction between the two were observed on PT and PF. However, there was a significant effect of time (*p* < 0.05) on HRT. The HRT was significantly decreased at POST-15 and POST-30, as previously described for the single twitch.

### Train 5 Hz

**Fig. 4:**
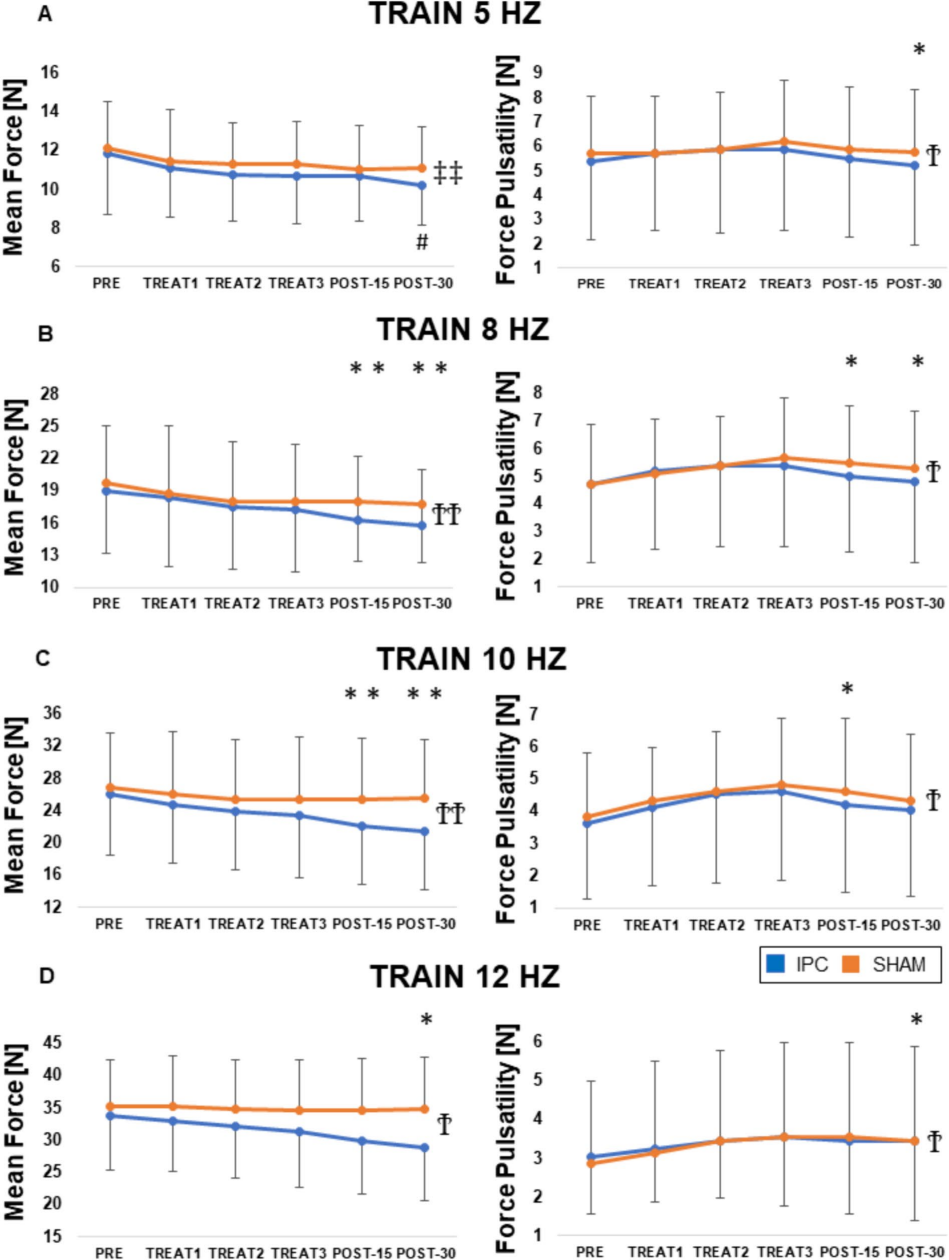
Sub-tetanic contractions. Average effect of IPC (black) and SHAM (gray) treatments on mean force and pulsatility during electrically stimulated contractions at different frequencies. ^Ϯ^ = main effect of time, *p* < 0.05; ^ϮϮ^ = main effect of time, *p* < 0.01; ^‡^ = time × group interaction, *p* < 0.05; ^‡‡^ = time × group interaction, *p* < 0.01; ^*^ = PRE vs. POST, *p* < 0.05; ^**^ = PRE vs. POST, *p* < 0.01; ^#^ = significantly different from PRE, only for IPC

The mean force achieved during 5-Hz stimulation was neither affected by time nor by treatment, but there was a significant interaction between them (*p* < 0.01). Post hoc tests revealed a significant decrease in MF at POST-30 compared to baseline (from 10.2 to 11.9 N, *p* < 0.05), only for the IPC group. The main effect of time showed a statistically significant difference in the mean of PLS at POST-30 (*p* < 0.05). These results are depicted in Fig. 4A.

### Train 8 Hz

At 8-Hz stimulation frequency, we observed a significant effect of time (*p* < 0.01) on MF with no effect of treatment and no interaction. Mean force significantly decreased at POST-15 (16.3 N, *p* < 0.01) and POST-30 (15.8 N, *p* < 0.01) compared to baseline (19.0 N), in the IPC group only. We then investigated the train signals of 8 Hz of the PLS: the main effect of time showed a statistically significant difference in the mean of PLS at POST-15 and POST-30 (*p* < 0.05). These results are depicted in Fig. 4B.

### Train 10 Hz

At 10-Hz stimulation frequency, we observed a significant effect of time (*p* < 0.01) on MF with no effect of treatment and no interaction. Mean force significantly decreased at POST-15 (22.0 N, *p* < 0.01) and POST-30 (21.4 N, *p* < 0.01) compared to baseline (26.1 N), in the IPC group only. The main effect of time showed a statistically significant difference in the mean of PLS at POST-15 (*p* < 0.05).

### Train 12 Hz

At 12-Hz stimulation frequency, we observed a significant effect of time (*p* < 0.05) on PLS (*p* < 0.05) with no effect of treatment and no interaction. Mean force significantly decreased at POST-30 (28.81 N, *p* < 0.05) compared to baseline (33.71 N), in the IPC group only. The main effect of time showed a statistically significant difference in the mean of PLS at POST-30, (*p* < 0.05).

## Discussion

The present study aimed to explore the impact of IPC on the muscle contractile mechanism elicited by electrical stimulation. The findings revealed a significant progressive weakening of sub-tetanic contractions with time in both IPC and SHAM groups. Interestingly, this weakening is associated with a slight shortening of the twitch contraction (reduced HRT) rather than a decrease in peak force. Overall, IPC does not prevent this weakening effect and, in some cases, may exacerbate it.

Many studies have reported the beneficial effects of IPC on the performance of different sports, including running (Bailey et al. 2012), cycling (Patterson et al. 2015) and others (Marocolo et al. 2019; Salvador et al. 2016). The mechanisms underlying these improvements are unclear and may be ascribed to both systemic effects, including actions on the cardiovascular and central nervous system (Caru et al. 2019; Salvador et al. 2016), and local effects, taking place in the relevant muscles (Salvador et al. 2016). More specifically, an increase in muscle force has been reported in different experimental conditions, involving a reduced number of recruited muscles and joints. This effect was observed in both aerobic (Bailey et al. 2012) and anaerobic exercise (Rodrigues et al. 2023; Telles et al. 2022a), at exhaustion (Allois et al. 2023) and non-fatigue conditions (Telles et al. 2022b).

The ergogenic effects of IPC, specifically taking place in skeletal muscles, can be related to three different aspects: vascular changes with improvement of blood perfusion, metabolic changes with improvement in O2 utilization and changes in the molecular contractile mechanisms with improvement in the force development capacity (O’Brien and Jacobs 2021, 2022). While the first two mechanisms may improve muscle performance in long-lasting and fatiguing contraction, they are unlikely to explain the force increase during short efforts in the non-fatigued muscles. Effects in this latter condition could only be explained by a change in muscle contractility or a change in the motor drive. By investigating electrically stimulated contractions, we have ruled out any possible IPC effect on the central nervous system, potentially affecting the motor drive. With this approach, the results did not evidence any ergogenic effect.

Single twitches evoked by a single stimulation pulse are the simplest model of contraction, but may be affected by the extent of initial tension of the muscle. Paired stimuli (doublets) or short stimulation bursts are often also considered, as they provide a more robust manifestation of muscle contractility (Roatta and Farina 2011). In addition, sub-tetanic contractions, as obtained by constant frequency stimulation (trains), may be helpful in highlighting changes in the summation process of single twitches. In fact, a small decrease/increase in HRT and twitch duration may result in prominent decrease/increase in the achieved force and in a possible increase/decrease in force pulsatility, due to reduced/increased twitch summation (Crivelli et al. 2013; Roatta and Farina 2011). Our results align with this principle: effects on burst stimulation confirm the general weakening trend in both the IPC and the SHAM groups observed with single twitches, namely, the reduction in HRT, revealing a slight shortening of the force pulse. Accordingly, the mean force achieved during train stimulation decreased over time in both groups, reflecting the reduced twitch summation. Notably, at 5 Hz, the mean force decrease was even larger in the IPC group compared to SHAM, suggesting a weakening rather than an ergogenic effect of IPC. It is also worth emphasizing that the observed effects cannot be attributed to decreased effectiveness of the electrical stimulation, as no decrease in the sEMG M-wave was detected throughout. In addition, local blood volume and tissue oxygenation were also maintained throughout the experimental session, indicating absence of relevant changes in blood perfusion and metabolism. To our knowledge, only one research group introduced electrically stimulated contractions in assessing the effects of IPC (Halley et al. 2018, 2019a, b; Marshall et al. 2020). The authors delivered single twitches before and during MVC to assess changes in voluntary activation (superimposed twitch technique). Consistent with our findings, they also reported a lack of ergogenic IPC effects on the twitch force and a general weakening trend in both IPC and SHAM groups.

The present results are only in apparent contrast with those reporting increased muscle force in non-fatigued conditions. Rodrigues et al. (2023) observed that IPC increased by ~ 2.4-kg (~ 2%) one repetition maximum (1-RM) force in bench-press exercise with respect to baseline, while the 1.8-kg increase in the SHAM group did not reach significance (but the IPC–SHAM difference was not explicitly tested). Admittedly, the effect could be attributed to increased motor drive rather than increased contractility. Along the same line, Telles et al. (2022a) report that IPC treatment (at both arms alternatively), performed at both high (220 mmHg) and low pressure (20 mmHg: which is usually identified as a “SHAM” treatment), effectively increased the maximum strength (1 RM) in various exercises, including bench press and leg extension, in both groups, compared to the control group (no treatment). Similar effects (increased strength in maximal handgrip and other tasks) were observed by the same group in elderly women (Telles et al. 2022b). Again, these data suggest the occurrence of a systemic effect, which increases the voluntary motor drive to the muscles. The authors hypothesized that the low-pressure treatment could have activated the same mechanisms activated by blood flow restriction (although blood flow was not measured) (Queiros et al. 2023). However, the possibility exists that the placebo effect is not adequately prevented by a 20-mmHg compressive treatment. On the other hand, many studies failed to evidence a significant IPC-induced increase in force in non-fatigued muscles, e.g., in the same bench-press exercise (Valenzuela et al. 2021).

## Limitations

A limitation of the present study is that it was conducted on a small hand muscle, which has no crucial role in most sports activities. However, the AP presents several advantages, including the fact that it has no agonist muscles, and it can be easily monitored with sEMG and electrically stimulated via the ulnar nerve. We have no reason to expect a peculiar IPC response from this muscle. Therefore, we are confident that the results of the present study may apply to skeletal muscles in general. Due to the fact that bipolar, rather than monopolar, EMG recording was performed, we could not implement the separate analysis of the first and second phases of the compound action potential (Rodriguez-Falces and Place 2014, 2016), which could reveal possible alterations of membrane excitability in muscle fibers, introduced by IPC and/or SHAM treatments. Whether the observed decrease in twitch force with time is due to alteration of membrane excitability remains to be ascertained. A further limitation is that a 3 × 5 IPC protocol was employed. Although this pattern proved effective in improving muscle performance in several previous investigations (Salvador et al. 2016; Marocolo et al. 2019; Allois et al. 2023), we cannot exclude that protocols of longer duration could have produced different effects.

## Conclusion

In conclusion, the present results support the notion that no increase in the force capacity is introduced by IPC in skeletal muscles and that possible ergogenic effects in non-fatigued muscles are due to changes in the motor drive mediated by placebo or remote effects on the central nervous system. The present observations do not negate the possible ergogenic effects of IPC in muscles engaged in endurance exercise and fatigue conditions.

## Perspective

Ergogenic effects of IPC have been demonstrated in various sports disciplines; however, the underlying mechanisms are unclear, and the placebo effect has also been recognized as a relevant confounding factor. Clarifying the involved mechanisms may help to tailor IPC treatments to the relevant activities and conditions. We here evidenced that IPC does not potentiate contractility in non-fatigued muscles, supporting the concept that ergogenic effects in these conditions may only result from increased motor drive. In this respect, the present results have implications in impulsive motor activity and emphasize the need to account for the placebo effect when investigating IPC effects in general tasks based on voluntary muscle activation.

## Data Availability

Data will be made available upon reasonable request.

## Abbreviations

ABP: Arterial blood pressure
ABPd: Arterial blood pressure—diastolic
ABPs: Arterial blood pressure—systolic
ANOVA: Analysis of variance
AP: Adductor pollicis
ES: Electrically stimulated
HRT: Half-relaxation time
IPC: Ischemic pre-conditioning
MF: Mean force
MVC: Maximum voluntary contraction
M-wave: Average curve calculated for the sEMG signal
NIRS: Near-infrared spectroscopy
PF: Peak force
PLS: Pulsatility
POST-15: 15 Min after the treatment
POST-30: 30 Min after the treatment
PT: Time to peak
sEMG: Surface electromyographic signal
AmpliPP: Peak-to-peak amplitude of the M-wave
SSL: Supramaximal stimulation level
TEMP: Temperature
THI: Tissue hemoglobin index
TOI: Tissue oxygenation index
